# Minimally invasive left internal mammary artery harvesting techniques during the learning curve are safe and achieve similar results as conventional LIMA harvesting techniques

**DOI:** 10.1186/s13019-022-01961-0

**Published:** 2022-08-24

**Authors:** Matiullah Masroor, Chunyang Chen, Kang Zhou, Xianming Fu, Umar Zeb Khan, Yuan Zhao

**Affiliations:** 1 Department of Cardiovascular Surgery, The Second Xiangya Hospital of Central South University, 139 Renmin Middle Rd, Changsha, 410011 China; 2 Department of Cardiothoracic and Vascular Surgery, Amiri Medical Complex, Qargha Rd, Afshar, Kabul, Afghanistan; 3 Department of Surgery, Xiangya Hospital of Central South University, Xiangya Rd, Changsha, 410000 China

**Keywords:** Internal mammary artery, Minimally invasive surgery, Harvesting, CABG, Learning curve

## Abstract

**Background:**

Internal thoracic arteries (ITAs) are considered to be the standard conduits used for coronary revascularization. Recently minimally invasive procedures are performed to harvest ITAs. The aim of this retrospective cohort study is to observe the effect and safety of less invasive LIMA harvesting approaches in the learning curve compared to conventional harvesting.

**Methods:**

We retrospectively analyzed the data of 138 patients divided into three different groups based on the LIMA harvesting techniques: conventional sternotomy LIMA harvesting, CSLH (n: 64), minimally invasive direct LIMA harvesting, MIDLH (n: 42), and robotic-assisted LIMA harvesting, RALH (n: 32). The same 138 patients were also divided into sternotomy (n: 64), and non-sternotomy (n: 74) groups keeping both MIDLH and RALH in the non-sternotomy category. Parameters associated with LIMA’s quality and some other perioperative parameters such as harvesting time, LIMA damage, perioperative myocardial infarction, ventilation time, 24 h drainage, ICU stay, hospital mortality, computed tomographic angiography (CTA) LIMA patency on discharge, and after one year were recorded.

**Results:**

The mean LIMA harvesting time was 36.9 ± 14.3, 74.4 ± 24.2, and 164.7 ± 51.9 min for CSLH, MIDLH, and RALH groups respectively (*p* < 0.001). One patient 1/32 (3.1%) in the RALH group had LIMA damage while the other two groups had none. One-month LIMA CTA patency was 56/57 (98.2%), 34/36 (94.4%), and 27/27 (100%) (*p* = 0.339), while 1 year CTA patency was 47/51 (92.1%), 30/33 (90.9%), and 24/25 (96%) for CSLH, MIDLH, and RALH groups respectively (*p* = 0.754). In the case of sternotomy vs non-sternotomy, the LIMA harvesting time was 36.9 ± 14.3 and 113.6 ± 59.3 min (*p* < 0.001). CTA patency on discharge was 56/57 (98.2%) and 61/63 (96.8%) (*p* = 0.619), while 1 year CTA patency was 47/51 (92.1%) and 54/58 (93.1%) (*p* = 0.850) for sternotomy vs non-sternotomy groups.

**Conclusion:**

Minimally invasive left internal mammary artery harvesting techniques during the learning curve are safe and have no negative impact on the quality of LIMA. Perioperative outcomes are comparable to conventional procedures except for prolonged harvesting time. RALH is the least invasive and most time-consuming procedure during the learning curve. These procedures are safe and can be performed for selected patients even during the learning curve.

## Introduction

In recent decades there are less invasive and sternal sparing procedures available for CABG surgery such as minimally invasive direct coronary artery bypass surgery (MIDCAB), endoscopic assisted coronary artery bypass surgery (Endo ACAB), robotic-assisted coronary artery bypass surgery (RACAB), and totally endoscopic coronary artery bypass surgery (TECAB) [1, 2]. After these minimally invasive options are available for the treatment of coronary artery diseases, the patient’s demands for receiving these minimally invasive procedures are increasing too.

As the patency of the left anterior descending artery (LAD) determines survival after surgery, and the left internal mammary artery (LIMA) is considered the gold standard conduit for revascularization [3, 4]. It is routine practice in cardiac surgery to graft LIMA to the LAD to achieve these benefits [5, 6]. LIMA harvesting is considered one of the most important steps in coronary artery bypass surgeries after the documented survival benefits of LIMA to LAD grafting by Loop et al. [7]. To achieve this goal of minimally invasive CABG, LIMA has to be harvested with minimally invasive techniques. After classical sternotomy LIMA harvesting techniques [8], LIMA is now also harvested with mini anterolateral thoracotomy under direct vision [9], thoracoscopic harvesting of LIMA [10], and robotic-assisted LIMA harvesting [11]. Regardless of the anastomosis techniques, surgeons are trying to harvest the LIMA with minimally invasive techniques to meet the demands of less invasive procedures.

After these different harvesting techniques are introduced to the surgical arena, it is important to ensure the safety and efficacy of these minimally invasive procedures, and the quality of LIMA to be used for revascularization after harvesting with these different harvesting techniques. Studies have been performed to compare the outcomes of these MICABG to the conventional CABG and the results are satisfactory [12, 13], but less amount of work is available focusing on the quality of LIMA during harvesting of these different harvesting techniques, especially during the learning curve. In the present study we aimed to see the effects of different LIMA harvesting techniques during the learning curve on LIMA’s quality in terms of LIMA damage, perioperative myocardial infarction (PMI), computed tomographic angiography (CTA) LIMA’s patency on discharge and after 1 year, and other perioperative parameters such as harvesting time, conversion to sternotomy, need of CPB, reoperation for bleeding, ICU stay, postoperative drainage, and hospital mortality were recorded to observe the safety and efficacy of these minimally invasive procedures. To the best of our knowledge, it is the first study comparing these three different LIMA harvesting techniques and their effect on LIMA’s integrity during the learning curve.

## Methods

The study was conducted in accordance with the Declaration of Helsinki (as revised in 2013). The study was approved by the institutional ethics committee of the Second Xiangya Hospital of Central South University and individual consent for this retrospective analysis was waived.

### Patients

**Fig. 1:**
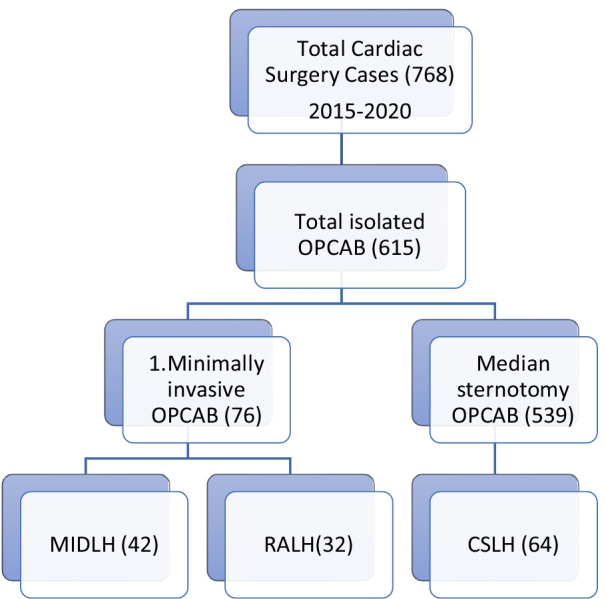
Study design shows how the patients were classified into three groups. *OPCAB* off-pump coronary artery bypass, *MIDLH* minimally invasive direct LIMA harvesting, *RALH* robotic-assisted LIMA harvesting, *CSLH* conventional sternotomy LIMA harvesting

**Fig. 2:**
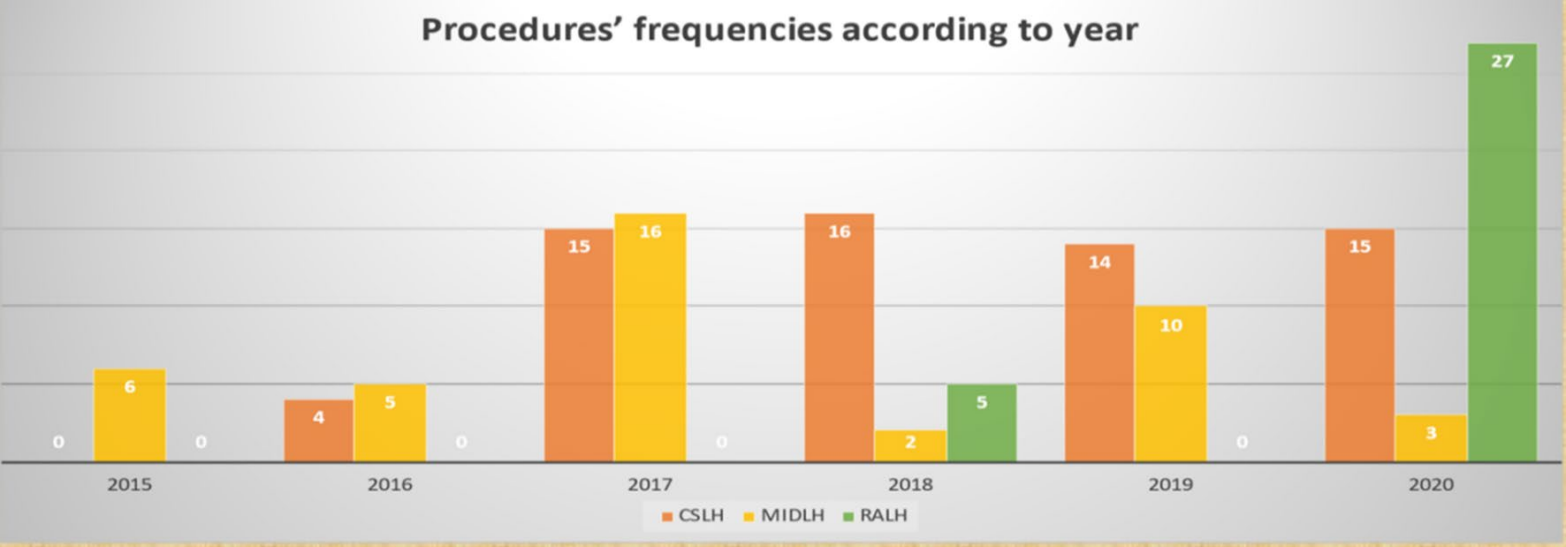
Numbers of CSLH, MIDLH, and RALH cases performed with respect to each year from 2015 to 2020

**Fig. 3:**
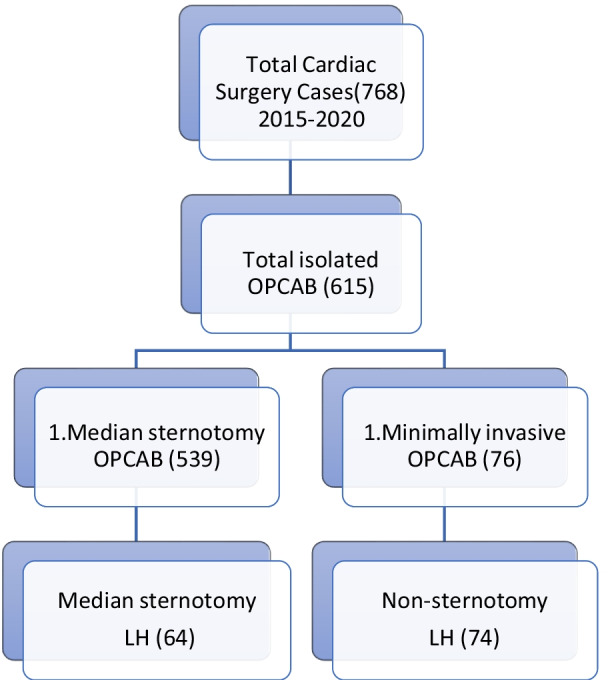
Study design shows how the patients were classified into sternotomy versus non-sternotomy groups. *OPCAB* off-pump coronary artery bypass, *MIDLH* minimally invasive direct LIMA harvesting, *RALH* robotic-assisted LIMA harvesting, *CSLH* conventional sternotomy LIMA harvesting

The patient database of the Second Xiangya Hospital of Central South University was searched for the total cardiac surgeries done by a single surgical group from January 2015 to December 2020. A total of 768 cardiac surgeries were performed. On pump (with cardiopulmonary bypass) CABG surgeries, CABG surgeries with concomitant cardiac procedures, and surgeries other than CABG were excluded. 615 isolated Off-pump CABG surgeries were performed during the given period. 76 surgeries out of these 615 surgeries were minimally invasive CABG surgeries. We divided these patients into three groups based on different LIMA harvesting techniques. 42 patients’ LIMA was harvested through mini left anterolateral thoracotomy under the direct vision (MIDLH group), 32 patients’ LIMA was harvested with the help of the da Vinci robot system (RALH group), and the rest of the patients’ LIMA was harvested through conventional sternotomy approach from which only 64 patients (CSLH group) with the 1:2 were selected based on age, gender, height, weight, BMI etc. compared to the RALH group. Two cases in the minimally invasive group were thoracoscopic LIMA harvesting which were excluded. The total number of patients included in this study were 138 divided into three groups (CSLH n: 64), (MIDLH n: 42), and (RALH n: 32). The study design is given in Fig. 1. The frequencies of these procedures with respect to each year are given in Fig. 2. LIMA’s quality was only assessed by analyzing clinical data such as LIMA’s damage during harvesting, perioperative myocardial infarction (PMI) (according to the 4th universal definition of myocardial infarction type V within 48 h of surgery [14]), on discharge and 1 year LIMA’s patency by CTA, and other perioperative parameters such as LIMA’s harvesting time, conversion to sternotomy, need of CPB, reoperation for bleeding, 24 h postoperative chest tube drainage, total ICU stay, and hospital mortality were recorded to observe the safety and efficacy of these procedures. Because our focus was only on the LIMA graft, patients were considered negative for PMI if the cardiac biomarkers and ECG were positive for MI but CTA shows patent LIMA graft within 48 h after surgery. Another type of study design dividing the same 138 patients into sternotomy and sternal sparing techniques was also done. The sternotomy group (n: 64) and non-sternotomy group (n: 74) (containing patients of both MIDLH and RALH) were also analyzed for the same parameters and outcomes as given above for the three groups. The study design is shown in Fig. 3.

### Surgical techniques

#### Conventional sternotomy left internal mammary artery harvesting (CSLH)

**Fig. 4:**
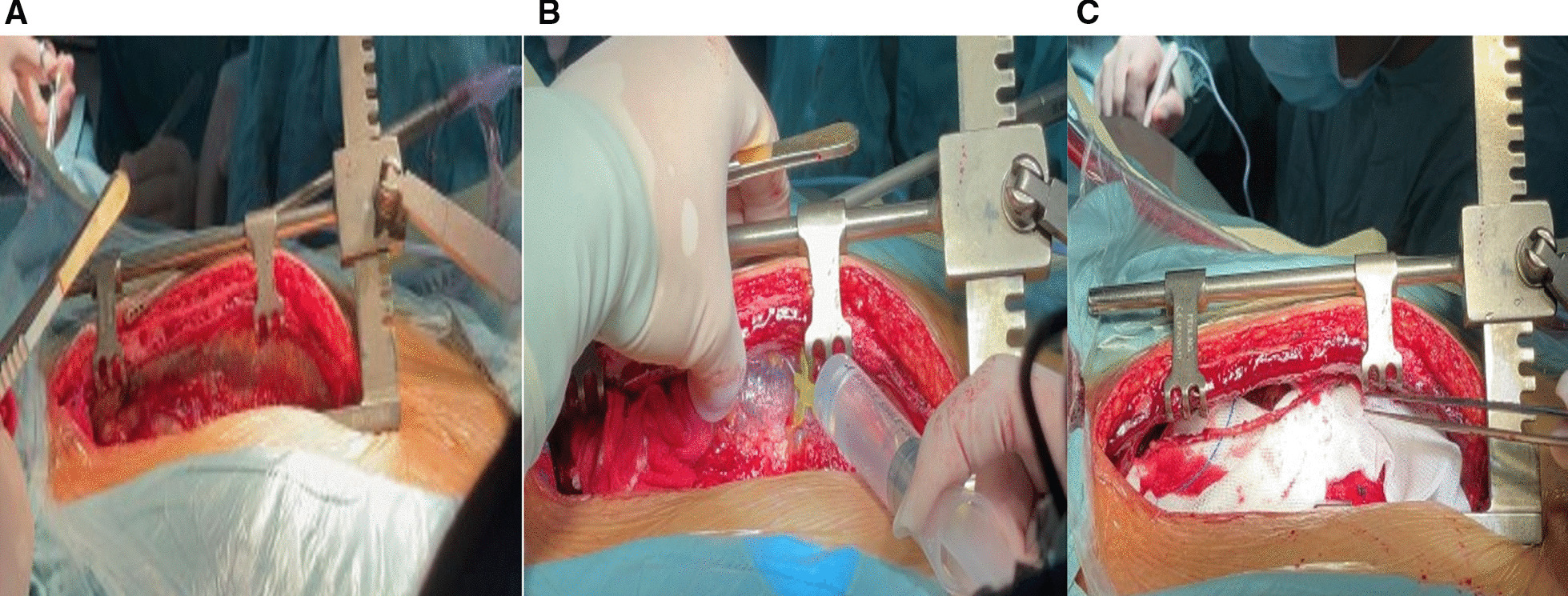
**A** The internal thoracic artery harvesting retractor has been applied and the left thorax is elevated. **B** The papaverine, normal saline, and diltiazem solution was injected into the endothoracic fascia before harvesting started. **C** LIMA after complete harvesting in skeletonized fashion

In this procedure after median sternotomy and hemostasis of the sternum and soft tissues, an IMA harvesting retractor (Fehling Surgical Instruments Inc., Karlstein, Germany) was used to elevate the left side of the chest as shown in Fig. 4. The table was then elevated to the chest level of the surgeon sitting on the right side of the patient to have a better vision of the LIMA site. The surgical table was moved away from the surgeon to better expose the LIMA and easily harvest it. A solution of 60 ml normal saline, 30 mg papaverine, and 10 mg diltiazem hydrochloride was injected into the endothoracic fascia along the plane of LIMA. The electrocautery was set at 15–18 W during harvesting. The harvesting would usually start at the proximal portion of LIMA just next to its origin from the left subclavian artery where LIMA was better exposed and extended inferiorly until LIMA bifurcation. The tip of the electrocautery was used as a dissector and care was taken to avoid touching the LIMA itself as much as possible. The branches were clipped with titanium clips and cut with a fine scissor. The LIMA was either harvested in a skeletonized fashion or pedicled fashion combined with its accompanying vein and tissues. The LIMA was kept perfused and intact to the systemic circulation until the time of anastomosis. Harvesting was done the same way as discussed by us somewhere else [15]. Before clamping the distal part of the LIMA, the patient received 1.5 mg heparin/kg of body weight intravenously to achieve an activated clotting time (ACT) of longer than 300 s. Most of the CSLH group patients received multiple grafts.

#### Minimally invasive direct left internal mammary artery harvesting (MIDLH)

**Fig. 5:**
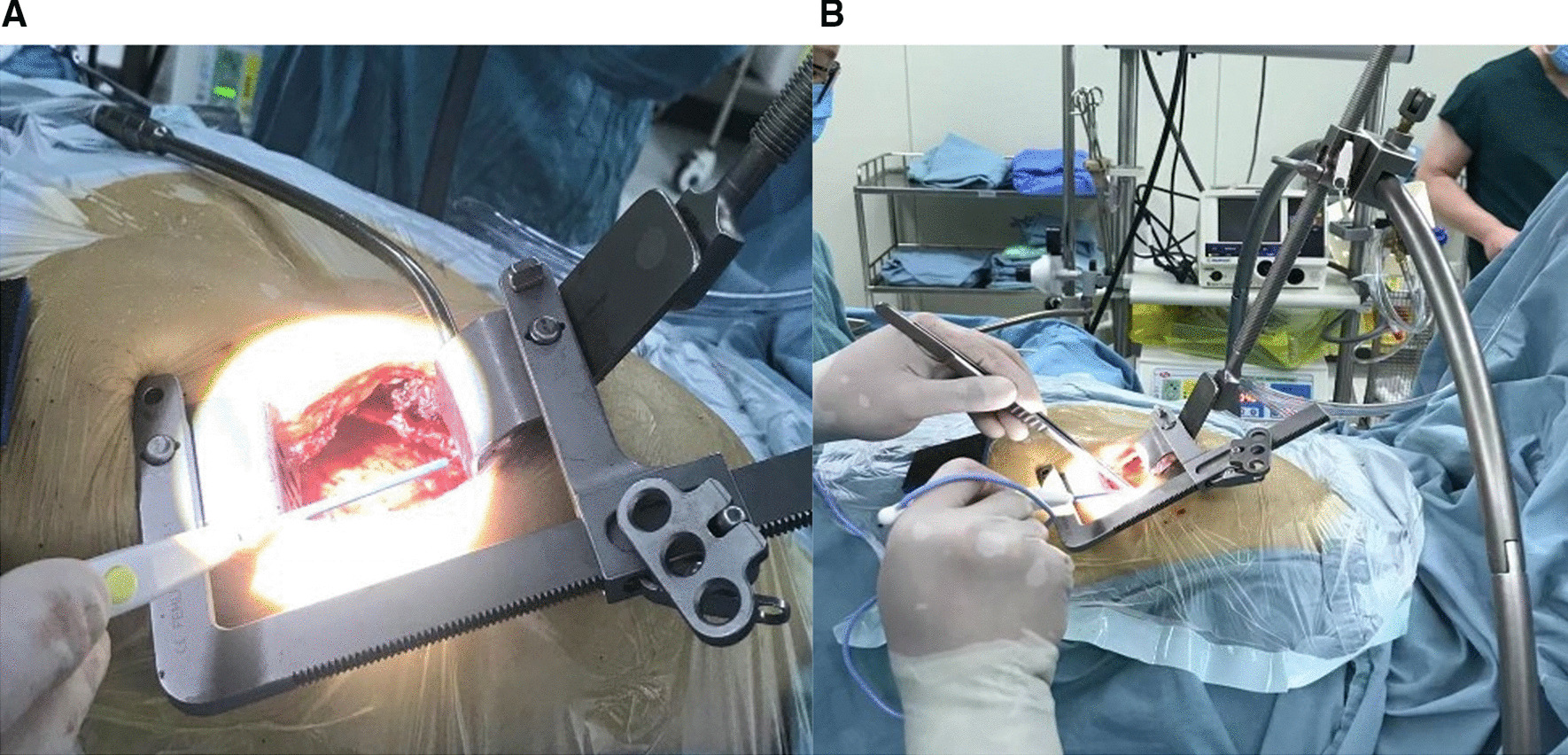
**A** The MICS CABG intercostal retractor for MIDLH procedure is seen. **B** The arch suspensory IMA retractor system is attached to the operating table and Thorac pro retractor attached to the middle of the arch

The patient was placed in a supine position with single lung ventilation with the help of a double lumen endotracheal intubation tube and the left side of the chest was elevated about 30–35 degrees. An anterolateral thoracotomy of 5–7 cm long was made in the 4th or 5th intercostal spaces 1/3rd to the right of the midclavicular line and 2/3rd to the left of the midclavicular line on the left side of the chest. In men, the incision was made just below the left nipple, and in a female under the left breast in the breast fold. The MICS CABG intercostal retractor with Thorac pro internal mammary artery harvesting retractor (Fehling Surgical Instruments Inc., Karlstein, Germany) was used to retract the ribs in combination with the arch suspensory internal mammary artery retractor system fixed to the operating table to lift the thorax as shown in Fig. 5. The left internal mammary artery was then harvested in a pedicled fashion from the lateral side as a mirror image to the CSLH technique under direct vision. The harvesting techniques were almost similar as discussed for classical sternotomy left internal mammary artery harvesting. All of the MIDLH group patients received a single graft (LIMA-LAD).

#### Robotic-assisted left internal mammary artery harvesting (RALH)

**Fig. 6:**
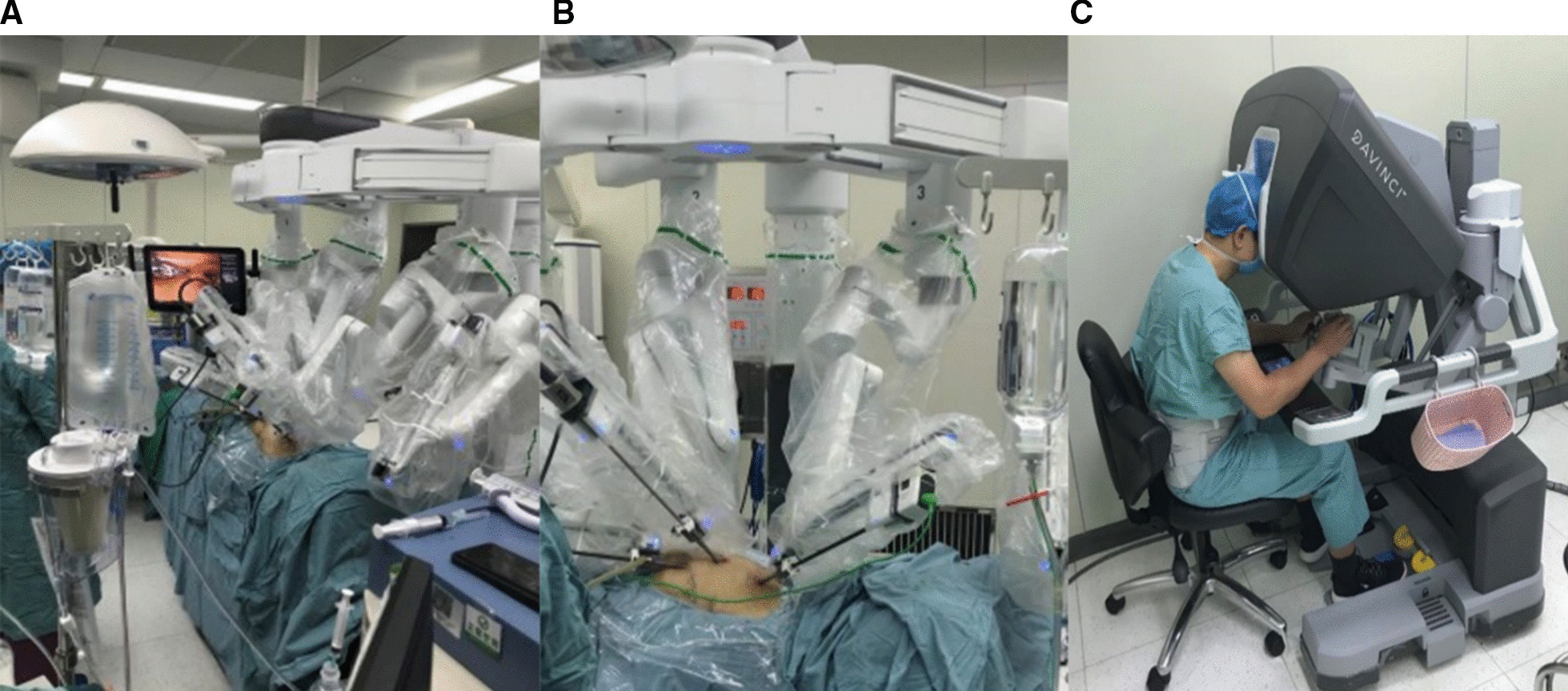
**A** Overall robotic system is seen docked to the patient. **B** Endo wrist instruments inserted through three ports into the left hemi thorax for RALH. **C** Surgeon sitting on surgeon’s console of da vinci robot system harvesting the LIMA

After placing the patient in a supine position with the left chest a little elevated and the left lung isolated with a double lumen endotracheal tube as explained above for MIDLH. The da Vinci robot system (Intuitive Surgical Inc., Sunnyvale, CA USA) was docked to the operating table. Three ports were inserted into the left thorax usually in the 3rd, 5th, and 7th intercostal space. The camera port was inserted into the 5th intercostal space in the anterior axillary line through a 5 mm incision. Care was taken not to damage the heart. After a warm CO_2_ insufflation to a pressure of 8 to 12 mm Hg, the other two instrument ports were inserted carefully under endoscopic guidance in the 3rd and 7th intercostal space. The spatula cautery was inserted in the 3rd intercostal space through a 5 mm incision in the mid to anterior axillary line. Micro bipolar forcep was inserted in another 5 mm incision made in the 7th intercostal space in the anterior axillary to the lateral clavicular line. The endo wrist monopolar spatula cautery was used by the right hand and endo wrist micro bipolar forcep was used by the left hand during harvesting. The Left internal mammary artery was unroofed from proximal to distal. The LIMA was harvested from the 1st to 6th rib in the beginning in skeletonized fashion and later on in pedicled fashion by the surgeon sitting away at the surgical console as shown in Fig. 6. The branches were clipped with an Endo clip and cut with scissors. The unroofing and harvesting of LIMA was performed with the help of Endo wrist instruments (Intuitive Surgical). All the RALH group patients received a single graft (LIMA-LAD).

### Statistical analysis

The data were analyzed by Statistical Package for Social Sciences for Windows, version 26 (SPSS) (statistical package for the social sciences Inc, Chicago IL, USA). The results for continuous variables are illustrated in mean plus standard deviation (SD) and the categorical variables are illustrated in number plus percentage (%). The alpha level of < 0.05 was considered statistically significant.

#### Three groups (CSLH/MIDLH/RALH) statistical analysis

One way analysis of variance (ANOVA) was used in this case. When there was a big difference in means and standard deviation, robust tests of equality of means with Brown-Forsythe and Welch were performed to make sure if there is any statistically significant difference. Post hoc analysis for statistically significant variables was done with Fisher’s least significant difference (LSD) test, but because our all-groups participants were not the same number so, Gabriel’s, and Hochberg’s GT2 tests were applied too to avoid any type of error.

#### Two groups (sternotomy vs. non-sternotomy) statistical analysis

Unpaired student t-test was used for the comparison of continuous variables. Pearson’s chi-square (X^2^) test was used for categorical nominal variables and fisher’s exact test was used in case of small expected variable frequencies (cell size less than five).

## Results

### Three groups (CSLH/ MIDLH/RALH) analysis

Preoperative demographical data were comparable in all groups with no statistically significant difference except hypertension and BMI. The mean age was 61.6 ± 10.6, 60.5 ± 10.8, and 61.5 ± 10.5 years for CSLH, MIDLH, and RALH groups respectively (*p* = 0.873). The male gender was 52 (81.2%), 33 (78.6%), and 26 (81.2%) for CSLH, MIDLH, and RALH groups respectively (*p* = 0.937). There were 23 (35.9%) diabetic patients in CSLH, 13 (30.9%) in MIDLH, and 9 (28.1%) in RALH group (*p* = 0.721). The number of smokers were 29 (45.3%) in CSLH, 15 (35.7%) in MIDLH, and 12 (37.5%) in RALH group (*p* = 0.573) but the difference did not reach statistically significant level. The patients who were having a history of kidney disease were 5 (7.8%), 3 (7.1%), and 0 (0%) in CSLH, MIDLH, and RALH groups respectively (*p* = 0.279). Hypertension was a statistically significant variable with 49 (76.6%), 21 (50%), and 24 (75%) patients for CSLH, MIDLH, and RALH respectively with overall (*p* = 0.010). On post hoc analysis CSLH vs MIDLH (*p* = 0.004), MIDLH vs RALH (*p* = 0.021), and CSLH vs RALH was not statistically significant. BMI was also significant during analysis with 25.4 ± 3.0, 23.8 ± 3.0, and 24.6 ± 2.7 kg/m^2^ for CSLH, MIDLH, and RALH respectively with overall (*p* = 0.022), CSLH vs MIDLH (*p* = 0.006), MIDLH vs RALH, and CSLH vs RALH were not statistically significant. HCR procedures were 10 (23.8%) in MIDLH, and 6 (18.7%) in RALH group. The preoperative demographical and clinical data of the three different groups are depicted in Table 1.

**Table 1:** Preoperative demographical and clinical data for CSLH, MIDLH, and RALH groups

Variables	CSLH (n = 64)	MIDLH (n = 42)	RALH (n = 32)	*p* value
Age (years)	61.6 ± 10.6	60.5 ± 10.8	61.5 ± 10.5	0.873
Male gender (%)	52 (81.2%)	33 (78.6%)	26 (81.2%)	0.937
Height (cm)	163.4 ± 6.9	163.4 ± 7.4	164.6 ± 7.4	0.669
Weight (kg)	68 ± 10.3	65 ± 10.9	68.3 ± 10.1	0.276
BMI (Kg/m^2^)	25.4 ± 3.0	23.8 ± 3.0	24.6 ± 2.7	0.022
Hx of hypertension (%)	49 (76.6%)	21 (50%)	24 (75%)	0.010
Hx of DM (%)	23 (35.9%)	13 (30.9%)	9 (28.1%)	0.721
Hx of hyperlipidemia (%)	9 (14.1%)	5 (11.9%)	5 (15.6%)	0.898
Hx of smoking (%)	29 (45.3%)	15 (35.7%)	12 (37.5%)	0.573
LVEF (%)	63.1 ± 8.5	63 ± 9.2	67.2 ± 7.7	0.060
LVEDD (mm)	48.0 ± 5.6	46.9 ± 5.3	47.8 ± 5.1	0.573
Hx of renal impairment (%)	5 (7.8%)	3 (7.1%)	0 (0%)	0.279
Hx of COPD (%)	0 (%)	2 (4.8%)	0 (0%)	0.099
Hx of CVD (%)	8 (12.5%)	8 (19%)	5 (15.6%)	0.660
Hx of PVD (%)	4 (6.2%)	3 (7.1%)	1 (3.1%)	0.752
HCR (%)	8 PCI (12.5)	10 (23.8%)	6 (18.7%)	NA

*Hx* history, *BMI* body mass index, *DM* diabetes mellitus, *LEVEF* left ventricular ejection fraction, *LEVEDD* left ventricular end diastolic diameter, *COPD* chronic obstructive pulmonary disease, *CVD* cerebrovascular disease, *PVD* peripheral vascular disease, *HCR* hybrid coronary revascularization, *PCI* percutaneous coronary intervention

In perioperative and postoperative outcomes variables, there was no need for CPB, re-exploration for bleeding, no perioperative MI, and no in-hospital mortality in all groups. LIMA harvesting time was statistically significant 36.9 ± 14.3, 74.4 ± 24.2, 164.7 ± 51.9 min for CSLH, MIDLH, and RALH groups respectively with overall (*p* < 0.001) and comparison of all three groups on post hoc analysis, all the *p* values were also < 0.001. The 24 h postoperative chest tube drainage was also significant with 578.8 ± 258.3 ml in CSLH, 451.1 ± 399.2 ml in MIDLH, and 285.3 ± 313.0 in the RALH group with overall (*p* < 0.001). CSLH versus MIDLH (*p* = 0.047), CSLH versus RALH (*p* < 0.001), and MIDLH versus RALH (*p* = 0.030) on post hoc analysis. ICU stay was with 50.7 ± 36.1 h in the CSLH group, 34.9 ± 27.2 h in the MIDLH group, and 37.1 ± 25.8 h in the RALH group with an overall (*p* = 0.024). CSLH versus MIDLH (*p* = 0.013), CSLH versus RALH (*p* < 0.050), and MIDLH versus RALH was not statistically significant on post hoc analysis. Ventilation time was 17.3 ± 19.1 h in CSLH, 9.9 ± 12.6 h in MIDLH, and 9.2 ± 9.4 h in the RALH group with overall (*p* = 0.017), CSLH versus MIDLH (*p* = 0.018), CSLH versus RALH (*p* < 0.019), and MIDLH versus RALH was not statistically significant. There was one patient in the RALH group in which LIMA damage occurred. The LIMA was found to have no flow after trimming at the distal end after the completion of harvesting, which could have most probably been damaged by the cautery heat. We could not find the exact reason of the damage. Papaverine was sprayed and then intra-luminally injected but the flow could not resume. The surgery was carried out through a MIDCAB incision with a radial artery used as a conduit. There was no LIMA damage in any other group. In MIDLH and RALH groups all harvesting were completed without conversion to median sternotomy. CTA patency on discharge was 56/57 (98.2%) with stenosis of one graft, 34/36 (94.4%) with one stenosis of the graft and anastomosis each, and 27/27 (100%) for CSLH, MIDLH, and RALH respectively (*p* = 0.339). The CTA was performed only for 57/64 (89.1%), 36/42 (85.7%), and 27/31 (87.1%) patients for CSLH, MIDLH, and RALH groups respectively. One year CTA follow up was completed only for 51/64 (79.7%), 33/42 (78.6%), and 25/31 (80.6%) of patients and the patency rate was 47/51 (92.1%), 30/33 (90.9%), and 24/25 (96%) for CSLH, MIDLH, and RALH respectively (*p* = 0.754). Stenosis of the graft in two patients, graft occlusion in one, and anastomosis occlusion in one patient in the CSLH group, stenosis of the graft in two patients and anastomotic stenosis in one patient in MIDLH groups, and stenosis of the graft in one patient in RALH group at 1 year CTA was found. Neither CTA patency on discharge nor 1 year CTA patency reached a statistical significance level. The perioperative and postoperative outcomes of the three groups are given in Table 2.

**Table 2:** The peri- and postoperative outcomes for CSLH, MIDLH, and RALH groups

Variables	CSLH (n = 64)	MIDLH (n = 42)	RALH (n = 32)	*p* value
LIMA harvesting time (min)	36.9 ± 14.3	74.4 ± 24.2	164.7 ± 51.9	< 0.001
LIMA damage (%)	0 (0%)	0 (0%)	1 (3.1%)	0.192
Conversion to sternotomy (%)	NA	0 (0%)	0 (0%)	NA
Need of CPB (%)	0 (0%)	0 (0%)	0 (0%)	1.000
Post OP 24 h drainage (ml)	578.8 ± 258.3	451.1 ± 399.2	285.3 ± 313.0	< 0.001
Re-exploration for bleeding (%)	0 (0%)	0 (0%)	0 (0%)	1.000
Ventilation time (h)	17.3 ± 19.1	9.9 ± 12.6	9.2 ± 9.4	0.017
Total ICU stay (h)	50.7 ± 36.1	34.9 ± 27.2	37.1 ± 25.8	0.024
Perioperative MI (%)	0 (0%)	0 (0%)	0 (0%)	1.000
In hospital mortality (%)	0 (0%)	0 (0%)	0 (0%)	1.000
CTA patency on discharge (%)	56/57 (98.2%)	34/36 (94.4%)	27/27 (100%)	0.339
One year CTA patency (%)	47/51 (92.1%)	30/33 (90.9%)	24/25 (96%)	0.754

Continuous data were shown as mean ± SD and categorical data were shown as number plus %. One way ANOVA with Welch robust test for equality of means was applied for statistical analysis. Post hoc analysis was done with Fisher's LSD and Gabriel's tests

*CPB* cardiopulmonary bypass, *MI* myocardial infarction, *CTA* computed tomographic angiography

### Two groups (sternotomy vs. non-sternotomy) analysis

The mean age was 61.6 ± 10.6 and 61 ± 10.6 years (*p* = 0.757) for sternotomy vs non-sternotomy groups. Male gender was 52 (81.2%) for sternotomy and 59 (79.7%) for non-sternotomy group (*p* = 0.822). The number of smokers and diabetics were 29 (45.3%) and 27 (36.5%) (*p* = 0.292), and 23 (35.9%) and 22 (29.7%) (*p* = 0.438) for sternotomy versus non-sternotomy groups respectively. But these variables did not reach a statistically significant level. The statistically significant parameters were BMI and hypertension similar to the three groups analysis as mentioned above. The number of hypertensive patients in sternotomy and non-sternotomy were 49 (76.6%) and 45 (60.8%) with (*p* = 0.048) respectively. The sternotomy group had a higher mean BMI value of 25.4 ± 3.0 kg/m^2^ compared to the non-sternotomy group 24.1 ± 2.9 kg/m^2^ with (*p* = 0.013). The number of COPD patients was 0 (0%) and 2 (2.7%) in sternotomy vs non-sternotomy groups (*p* = 0.499). The baseline parameters of both groups are mentioned in Table 3.

**Table 3:** Preoperative demographical and clinical data of sternotomy and non-sternotomy groups

Variables	Sternotomy (n = 64)	Non-sternotomy (n = 74)	*p* value
Age (years)	61.6 ± 10.6	61 ± 10.6	0.757
Male gender (%)	52 (81.2%)	59 (79.7%)	0.822
Height (cm)	163.4 ± 6.9	163.9 ± 7.4	0.648
Weight (kg)	68 ± 10.3	66.4 ± 10.6	0.370
BMI (Kg/m^2^)	25.4 ± 3.0	24.1 ± 2.9	0.013
Hx of hypertension (%)	49 (76.6%)	45 (60.8%)	0.048
Hx of DM (%)	23 (35.9%)	22 (29.7%)	0.438
Hx of hyperlipidemia (%)	9 (14.1%)	10 (13.5%)	0.926
Hx of smoking (%)	29 (45.3%)	27 (36.5%)	0.292
LVEF (%)	63.1 ± 8.5	64.8 ± 8.8	0.239
LVEDD (mm)	48.0 ± 5.6	47.3 ± 5.2	0.449
Hx of renal impairment (%)	5 (7.8%)	3 (4.0%)	0.471
Hx of COPD (%)	0 (%)	2 (2.7%)	0.499
Hx of CVD (%)	8 (12.5%)	13 (17.6%)	0.409
Hx of PVD (%)	4 (6.2%)	4 (5.4%)	> 0.999

*Hx* history, *BMI* body mass index, *DM* diabetes mellitus, *LEVEF* left ventricular ejection fraction, *LEVEDD* left ventricular end diastolic diameter, *COPD* chronic obstructive pulmonary disease, *CVD* cerebrovascular disease, *PVD* peripheral vascular disease

The LIMA harvesting time was statistically significant in favor of Sternotomy 36.9 ± 14.3 and 113.6 ± 59.3 min (*p* < 0.001). The 24 h postoperative chest tube drainage, ventilation time, and ICU stay were also statistically significant in favor of non-sternotomy with 578.8 ± 258.3 and 380.7 ± 372 ml (*p* < 0.001), 17.3 ± 19.1 and 9.6 ± 11.3 h (*p* = 0.004), and 50.7 ± 36.1 and 35.8 ± 26.5 h (*p* = 0.006) for sternotomy vs non-sternotomy respectively. There was no need for CPB, re-exploration for bleeding, perioperative MI, and in-hospital mortality in both groups. On discharge, CTA was performed for 57/64 (89.1%) and 63/74 (85.1%) patients for sternotomy and non-sternotomy groups respectively. The CTA patency rate was 56/57 (98.2%) and 61/63 (96.8%) for sternotomy and non-sternotomy groups respectively. One year CTA patency was 47/51 (92.1%) and 54/58 (93.1%), and follow up was completed for 51/64 (79.7%) and 58/74 (78.4%) for sternotomy and non-sternotomy groups respectively. The perioperative and postoperative outcomes are tabulated in Table 4.

**Table 4:** Peri- and postoperative outcomes of sternotomy, and non-sternotomy groups

Variables	Sternotomy (n = 64)	Non-sternotomy (n = 74)	*p* value
LIMA harvesting time (min)	36.9 ± 14.3	113.6 ± 59.3	< 0.001
LIMA damage (%)	0 (0%)	1 (1.35%)	> 0.999
Need of CPB (%)	0 (0%)	0 (0%)	1.000
Post OP 24 h drainage (%)	578.8 ± 258.3	380.7 ± 372	< 0.001
Re-exploration for bleeding (%)	0 (0%)	0 (0%)	1.000
Ventilation time (h)	17.3 ± 19.1	9.6 ± 11.3	0.004
Total ICU stay (h)	50.7 ± 36.1	35.8 ± 26.5	0.006
Perioperative MI (%)	0 (0%)	0 (0%)	1.000
In hospital mortality (%)	0 (0%)	0 (0%)	1.000
CTA patency on discharge (%)	56/57 (98.2%)	61/63 (96.8%)	0.619
One year CTA patency (%)	47/51 (92.1%)	54/58 (93.1%)	0.850

Continuous data were shown as means ± SD and categorical data were shown as number plus %. Unpaired student's t test was used for continuous variables and Chi square test was used for categorical variables comparison. Fisher's exact test was used in case of a small cell size

## Discussion

In this study, we describe our very early experience of minimally invasive coronary artery bypass surgeries with a special focus on LIMA harvesting. In our hospital and probably in the whole province, these minimally invasive surgeries of MIDCAB and RACAB were first initiated by our team. Our first MIDCAB case was performed in the year 2015 and the first RACAB in the year 2018. So, the surgeries we performed till the end of the year 2020 were 42 MIDCAB and 32 RACAB, and these procedures are supposed to be the learning curve procedures. Though conventional sternotomy for CABG surgery can provide better exposure for LIMA harvesting, and LIMA can be harvested in full length which can be easily anastomosed to the distal part of LAD, but keeping the complications of median sternotomy (prolonged postoperative pain, longer recovery time, longer time to come back to normal daily activities, increase risk of superficial and deep sternal wound infection, increase risk of mediastinitis, etc.) in mind, MIDCAB was initiated. The LIMA harvesting techniques in MIDCAB are similar to that of conventional OPCAB except for sternal sparing small anterolateral thoracotomy and the use of a specialized retractor for exposure. So MIDLH procedure is actually a CSLH in limited space. These are the mirror image procedures of each other, in CSLH the LIMA is harvested medially while in MIDLH the LIMA is harvested laterally. This was the reason we took 1 ratio 2 patients of the CSLH group compare to the RALH group and not with comparison to the MIDLH group. Because we believe CSLH and MIDLH harvesting have very limited differences and the real difference lies in the comparison of CSLH with RALH. This was also the reason behind comparing the sternotomy group (CSLH), with the non-sternotomy group (MIDLH plus RALH).

A study by Stanislawski et al. comparing MIDCAB (n: 68) and OPCAB (n: 68) surgeries after propensity matching concluded that there was no in-hospital death in both groups, perioperative MI 1 (1.4%) and 2 (2.9%), reoperation for bleeding 4 (5.8%) and 2 (2.9%), 24 h chest drainage 440 ± 189 and 661 ± 372 ml, and ventilation time was 525 ± 314 and 569 ± 332 min for MIDCAB and OPCAB procedures respectively[16]. Another study by Raja et al. which include 508 MIDCAB and 160 OPCAB patients and preoperative demographical data were comparable. Their total operation time was 177 ± 32 and 141 ± 12 min, conversion to CPB was 0% and 1 (0.6%), re-exploration for bleeding was 16 (3.1%) and 4 (2.5%), and 30-day mortality was 10 (2.0%) and 4 (2.5%) for MIDCAB and OPCAB groups respectively. Conversion to sternotomy in the case of MIDCAB was 3 (0.6%), one was because of intra myocardial LAD, another with not enough length of LIMA, and the third one with LIMA damage [17]. A study from the third hospital of Peking university by Zhang et al. showed total surgery time of 152.0 ± 43.5 and 263.2 ± 52.4 min, postoperative ventilation time of 9.27 ± 5.14 and 24.92 ± 37.87 h, length of ICU stay 24.27 ± 17.25 and 59.13 ± 60.39 h, postoperative MI 2/300 (0.67%) and 2/355 (0.56%), re-exploration for bleeding 2/300 (0.67%), and 3/355 (0.85%), and 1 month mortality 1/300 (0.33%) and 3/355 (0.85%) for MIDCAB and OPCAB groups respectively [18]. Lima harvesting time in our study was 36.9 ± 14.3 and 74.4 ± 24.2 min, postoperative 24 h drainage 578.8 ± 258.3 and 451.1 ± 399.2 ml, ventilation time 17.3 ± 19.1 and 9.9 ± 12.6 h, and ICU stay of 50.7 ± 36.1 and 34.9 ± 27.2 h for CSLH and MIDLH groups respectively. There was no conversion to CPB, re-exploration for bleeding, perioperative MI, and hospital mortality in any group. Even though our main focus is on LIMA quality and harvesting related variables but all of the variables we have measured are in accordance with the studies mentioned above and other studies in the literature [19].

Harvesting of the LIMA through anterior thoracotomy (MIDLH) is associated with its unique challenges. It is hard to harvest the very distal part of the LIMA which may compromise the length of LIMA for anastomosis. Furthermore, excessive retraction and lifting of the chest wall is also associated with chest wall trauma and postoperative pain. To avoid these problems and add some length to the graft, like all other surgeons, an alternative to MIDLH which is RALH was initiated. RALH is a less invasive technique that harvests LIMA with the help of a robot. The short- and long-term outcomes of RACAB for revascularization surgery, which is in practice for the last two decades have been well documented. Because the procedure is performed without cardiopulmonary bypass same as MIDCAB and OPCAB, it can also avoid the associated risk of CPB such as inflammation, renal failure, and embolization [20]. The graft patency for RACAB and TECAB procedures has already been documented in the literature [21].

Halkos et al. published their result of 307 RACAB surgeries performed by two surgeons from October 2009 to September 2012. The conversion to sternotomy was 16 (5.2%), re-exploration for bleeding 7 (2.3%), postoperative MI 5 (1.6%), median ICU stay 1.0 days (range, 0–19), median ventilation time 2.0 h (range, 0–193), and 1 month mortality 4 (1.3%). Three out of 16 cases that converted to sternotomy were because of LIMA damage, inadequate length of LIMA, and LIMA dissection. On the follow up coronary angiography (CAG) 189/199 (95%) patients had patent graft (stenosis less than 50%) [22]. Another study showcased their 18 years of experience with RACAB. Their rate of conversion to sternotomy for any cause was 60 (9.9%). 32 (16%) in the first 200 cases, while 28 (6.9%) in the last 405 cases. Reoperation for bleeding 11 (1.8%), perioperative MI 8 (1.3%), ICU stay 1.2 ± 1.4 days, and death 2 (0.4%). 599/605 patients underwent CAG postoperatively and the LIMA to LAD graft patency was 97.4% [23]. A study by Gong et al. compared MIDCAB (n: 61) to RACAB (n: 71) and concluded that the midterm outcomes are comparable, but RACAB improves short term outcomes and midterm major adverse cardiac and cerebrovascular events free survival. The total operation time was 185.5 ± 49.3 and 220.8 ± 23.1 min, conversion to sternotomy was 2 (3.3%) and 3 (4.2%), ICU stay 35.2 ± 9.4 and 30.60 ± 8.7 h, reoperation for bleeding 1 (1.6%) and 1 (1.4%), MI 2 (3.3%) and 1 (1.4%), and 30-day mortality 1 (1.6%) and 0 (0%) for MIDCAB and RACAB respectively [24].

Fujita et al. reported their experience of the first 33 cases with RACAB which is almost the same number of cases as ours. The LIMA harvesting time in their study was 68 ± 13 min. They had 3 patients converted to sternotomy and the reason for conversion was bleeding from the LIMA in all three cases. By analysis, they found that the bleeding site in all three cases was adhesion to the second rib. They suggest that if the preoperative CT shows severe adhesion of LIMA to the second rib, harvesting should be stopped before the second rib especially, in the old age patient. The postoperative 3D CT evaluation of the graft resulted in 30/30 (100%) patent grafts [25]. Merwe et al. discussed the reason for conversion to sternotomy in the case of RACAB surgeries. They reviewed the data of 759 patients from 2002 to 2018. 30 (4.0%) patients converted to sternotomy. They divided conversion into two stages, early 12 (40%) and late 18 (60%) conversion. A conversion that occurred in the very beginning during the docking period or access to hemithorax was classified as early conversion, while conversion which occurred after safe access was achieved was classified as later conversion. The reasons for conversion were lung adhesion 11 (36.7%), inadequate lung isolation 1 (3.3%), ITA dysfunction 11 (36.7%), poor target vessel visualization 3 (10%), and ventricular perforation, arrhythmia, acute heart failure, and anastomosis dysfunction 1 (3.3%) each [26]. We also had one LIMA damage in the RACAB group but harvesting in that case was fully completed. After heparinization and anterior thoracotomy, the LIMA was found to have no flow while trimmed at the distal end. Vasodilators such as papaverine were sprayed and then intramurally injected but flow could not resume. The radial artery was harvested and aorta to LAD anastomosis was performed through MIDCAB incision. We believe that damage or dissection could have occurred because of the direct heat transferred to the intima during harvesting or heat transfer during hemostasis through a metallic clip that was applied very close to the LIMA. We firmly believe the damage was skills related. The LIMA harvesting time in our RACAB group was 164.7 ± 51.9 min, postoperative 24 h drainage of 285.3 ± 313.0 ml, ventilation time 9.2 ± 9.4 h, and total ICU stay of 37.1 ± 25.8 h. There was no conversion to sternotomy, need for CPB, re-exploration for bleeding, perioperative MI, and in-hospital mortality in our RACAB group. For LIMA grafts patency on discharge, we also got the same results with CTA on discharge as Fujita et al. [25], 27/27 (100%) of our grafts were patent. The graft patency at 1 year with CTA was also 96% with 24/25 grafts were patent.

Comparing sternotomy which consists of OPCAB (n: 234) patients to non-sternotomy which includes MIDCAB, Endo ACAB, and RACAB (n: 363) by Halkos et al. in 597 patients from January 2002 to June 2011 for LIMA to LAD revascularization. They reported mean total operative time of 2.28 ± 0.56 and 3.12 ± 1.05 h (*p* < 0.001), ICU length of stay of 50.3 ± 87.9 and 46.4 ± 82.5 h (*p* = 0.59), myocardial infarction 1 (0.4%) and 5 (1.4%) (*p* = 0.26), and 30-day mortality of 2 (0.9%) and 4 (1.1%) (*p* = 0.77) for sternotomy and non-sternotomy groups respectively [27]. In our study comparing sternotomy to non-sternotomy there was no perioperative MI and hospital mortality in any group. The LIMA harvesting time was 36.9 ± 14.3 and 113.6 ± 59.3 min (*p* < 0.001), total ICU stay of 50.7 ± 36.1 and 35.8 ± 26.5 h (*p* = 0.006), LIMA CTA patency on discharge of 56/57 (98.2%) and 61/63 (96.8%) (*p* = 0.619), and 1 year LIMA CTA patency of 47/51 (92.1%) and 54/58 (93.1%) (*p* = 0.850) for sternotomy (OPCAB) and non-sternotomy (MIDCAB and RACAB) groups respectively.

Because these techniques were first time practiced in our institution and new procedures always pertain to the learning curve. A study by Stanislawski et al. revealed that overall surgery time was significantly longer for the MIDCAB group compared to the OPCAB group, but with time after a learning threshold of 56 cases, the operating time of both groups was comparable [16]. Raja et al. also reported that operative time for MIDCAB was significantly longer than OPCAB, but they also believe that this time improves with experience. They compared their recent surgery time of MIDCAB with 10 years previous MIDCAB time which decreased significantly, 231 ± 14 min before 2007, and 132 ± 42 min in 2017 (*p* = 0.0001) [17]. A study by Une et al. also reported that their performance reached an acceptable level at the 66th case for single vessel MIDCAB [28]. Hemli et al. reported their experience with RACAB. They used their data to create a logarithmic learning curve for LIMA harvesting. The power function for LIMA harvesting defined the learning curve of 90% which means that during the entire course, a surgeon will experience an approximately 10% improvement in LIMA harvesting time with each doubling of the number of cases performed. They robotically harvested LIMA in 77 patients from January 2011 to July 2012. The mean LIMA harvesting time in their experience was 31.8 ± 10.1 min. The mean LIMA harvesting time in their first 10 cases was 39.00 ± 9.46 min and for the last 10 cases was 30.3 ± 6.9 min with (*p* = 0.03). They observed that all of their time variables decreased including LIMA harvesting time as the experience of the surgeon increased with most improvement seen in the first 20 cases [29]. Our LIMA harvesting time in the RALH group was considerably longer with a mean time of 164.7 ± 51.9 min. We also did not notice any significant improvement in LIMA harvesting time after crossing 20 procedures. The LIMA harvesting time in our first 10 RALH group was 181.9 ± 76.9 min and for the last 10 cases was 156.6 ± 36.6 min (*p* = 0.360). This may be influenced by the extensive practice and experience of a surgeon before starting his/her independent minimally invasive CABG surgeries which is nil in our case. More non-cardiac and cardiac robotic procedures may improve the skills of a surgeon which subsequently can improve the LIMA harvesting time. Recently in our institution, the LIMA harvesting with da Vinci robot is the only cardiac surgery procedure performed through a robot by a single surgeon (our team). So, we believe these are the reasons behind our prolonged LIMA harvesting time. However, a study by Eynde et al. who interpreted the results of their first 300 cases with RACAB described that the learning curve of their institution collectively got stabilized in the second tercile. Their results also described that about 50 surgeries are required to overcome the learning curve and get the desirable results of RACAB. They also achieved the most improvement in the surgical times during the first 100 cases [30]. We believe after performing 42 MIDCAB and 32 RACAB procedures we have not overcome the learning curve yet and the learning curve is not as short as 20 procedures.

As we discussed above, graft patency is the most important outcome which indicates long term survival and quality of life. Both invasive coronary angiography and computed tomographic angiography are commonly used for the analysis of graft patency postoperatively. Kiaii et al. achieved the graft patency for CSLH of 49/50 (98%) before the discharge, and Ruel et al. achieved 100% patency for MIDLH with CTA at 6 months for LIMA to LAD graft [8, 31]. Halkos et al., Patel et al., Kiaii et al., Giambrono et al., and Fujita et al. showed the patency rate for RALH before the discharge of 95%, 96.5%, 96.8%, 97.4%, and 100% respectively. All of their results were obtained from ICA except Fujita et al. which were from CTA [8, 22, 23, 25, 32]. Another study by Kiaii et al. showed the mean follow up of 9 months with angiography for the RALH group and the patency rate was 90.7% [33]. Our results for CTA graft patency on discharge was 56/57 (98.2%) for CSLH, 34/36 (94.4%) for MIDLH, and 27/27 (100%) for RALH groups. Similarly the 1 year CTA graft patency was 47/51 (92.1%), 30/33 (90.9%), and 24/25 (96%) for CSLH, MIDLH, and RALH respectively which are well in accordance with the literature [21].

We had a few limitations during the present study. The retrospective nature, single center, and small sample size make the study prone to bias. This study would have strengthened with the use of transient time flow metry (TTFM) during surgery to see the flow of graft before and after harvesting which is an important parameter for the quality of LIMA. Unfortunately, TTFM use is not a routine practice in our institution. Not 100% of patients underwent CTA on discharge and fewer could come for follow up CTA after 1 year which is another limitation of our study. A large sample size, multicentre, and prospective studies would address these issues.

## Conclusion

Left internal mammary artery harvesting with minimally invasive techniques such as MIDLH, and RALH during the learning curve are safe and have no negative impact on the quality and integrity of LIMA. These minimally invasive harvesting techniques have comparable outcomes compared to CSLH. The harvesting time in minimally invasive procedures is significantly longer during the learning curve which will improve over time. The graft patency is comparable to conventional harvesting techniques. RALH is the least invasive and most time-consuming LIMA harvesting approach during the learning curve. Therefore, minimally invasive LIMA harvesting can be performed safely during the learning curve in selected patients.

## Data Availability

All data generated and analysed during the current study are included in this published article.

## Abbreviations

ITA: Internal thoracic artery
LIMA: Left internal mammary artery
LAD: Left anterior descending artery
CABG: Coronary artery bypass grafting
CSLH: Conventional sternotomy LIMA harvesting
MIDLH: Minimally invasive direct LIMA harvesting
OPCAB: Off pump coronary artery bypass surgery
RALH: Robotic-assisted LIMA harvesting
DM: Diabetes mellitus
LVEF: Left ventricular ejection fraction
LVEDD: Left ventricular end diastolic diameter
COPD: Chronic obstructive pulmonary disease
PVD: Peripheral vascular disease
CVD: Cerebrovascular disease
CTA: Computed tomographic angiography
TTFM: Transient time flow metry
